# Perceived social support, insomnia, and psychological resilience: a chain mediation model of internet addiction in civil aviation cadets

**DOI:** 10.3389/fpsyt.2025.1698824

**Published:** 2025-11-13

**Authors:** Wenjia Chen, Bochuan Zhao, Haining Tu, Mingyu Liao, Xinan Zhang, Yuqi Su

**Affiliations:** 1 School of Physical Education, China University of Mining and Technology, Xuzhou, China; 2 School of Physical Education and Health, Guilin University, Guilin, China; 3 Department of Physical Training, Institute of Aviation Safety and Security, China Civil Aviation Flight Academy, Chengdu, China; 4 Student Affairs Office, Guilin No. 2 Technical School, Guilin, China

**Keywords:** perceived social support, insomnia, psychological resilience, internet addiction, chain mediation

## Abstract

**Purpose:**

Based on the stress-vulnerability model and protective-risk model, this study examines the mechanism by which perceived social support affects internet addiction among civil aviation flight cadets, focusing on testing the chain mediating role of insomnia and psychological resilience.

**Methods:**

Using a cross-sectional questionnaire survey, 1218 flight cadets (mean age approximately 18.8 years) were recruited through convenience sampling from a civil aviation flight college in China. Participants completed the Perceived Social Support Scale (PSS), Athens Insomnia Scale (AIS), Connor-Davidson Resilience Scale (CD-RISC), and Chinese Internet Addiction Scale-Revised (CIAS-R). Chain mediation analysis was performed using SPSS PROCESS macro Model 6 (Bootstrap resampling 5000 times, 95% confidence interval).

**Results:**

Significant correlations were found between all pairs of variables: perceived social support, insomnia, psychological resilience, and internet addiction (p < 0.01). Perceived social support was significantly negatively correlated with internet addiction (r=-0.34) and insomnia (r=-0.26), while significantly positively correlated with psychological resilience (r=0.65); insomnia was significantly positively correlated with internet addiction (r=0.30); psychological resilience was significantly negatively correlated with internet addiction (r=-0.44). Chain mediation analysis showed that perceived social support not only directly negatively predicted flight cadets’ internet addiction but also had indirect effects through insomnia and psychological resilience: on one hand, perceived social support indirectly reduced internet addiction by decreasing insomnia (r=-0.05); on the other hand, perceived social support indirectly reduced internet addiction by increasing psychological resilience (r=-0.21); additionally, perceived social support indirectly affected internet addiction through the chain mediating path of first reducing insomnia and then increasing psychological resilience (r=-0.01), and although the effect size of this chain mediation was small, it reached a significant level.

**Conclusion:**

Perceived social support has a significant inhibitory effect on internet addiction among civil aviation flight cadets, with insomnia and psychological resilience playing a partial chain mediating role. Enhancing flight cadets’ social support, improving their sleep status, and strengthening psychological resilience may alleviate the risk of internet addiction.

## Introduction

The widespread availability of the internet has made internet addiction a prevalent and important issue among young people (1, 2). Internet addiction typically refers to an individual’s pathological dependence on internet use, manifested as inability to control online time, leading to impaired daily life functioning (3, 4). Research shows that the incidence of internet addiction among young people in high-pressure environments such as college students and medical students is not negligible (5), and may be accompanied by problems such as sleep disorders, anxiety, and depression (6). Civil aviation flight cadets represent a special student group with strict training requirements and enormous pressure, whose physical and mental health directly affects flight safety. However, existing research on internet addiction mainly focuses on ordinary college students, with relatively little attention paid to flight cadets as a high-pressure pre-professional group. For instance, research has found that internet addiction and insomnia are highly correlated among medical students (7), and flight cadets similarly face strict schedules and study pressure, making them prone to related negative psychological conditions. According to the stress-vulnerability model, whether individuals experience adverse outcomes when facing stress depends on the interaction between risk factors and protective resources (8). For flight cadets, the high intensity of study and training and strict management constitute a continuous source of stress. If there is a lack of effective social support or individual psychological resources are vulnerable, stress may transform into sleep disorders and emotional problems, thereby inducing maladaptive coping such as internet addiction (9). However, with adequate social support and high psychological resilience, the impact of stress can be buffered, reducing the occurrence of maladaptive behaviors (9). The protective-risk model similarly points out that protective factors (such as social support, psychological resilience) can weaken the effect of risk factors (such as stress, sleep disorders) on adverse outcomes (10, 11). Based on these theories, this study focuses on two important protective factors—perceived social support and psychological resilience, as well as one risk factor reflecting stress impact—insomnia, to explore their relationships with internet addiction.

Perceived social support refers to the degree of support an individual subjectively feels from others and society, including support from family, friends, and significant others (12, 13). Adequate social support is considered an important external resource for coping with stress, helping to improve mental health and behavioral regulation. Wan et al. (2022) found that the mobile phone addiction level of teenagers could be reduced by increasing social support (14). Cui and Chi (2021) found that perceived social support could reduce adolescents’ tendency toward internet addiction by improving their psychological resilience and reducing post-traumatic stress symptoms (15). For individuals in high-pressure environments, such as military cadets and medical students, the buffering effect of social support is particularly crucial: it not only directly alleviates their psychological pressure but also prevents negative behaviors by enhancing their coping abilities. Therefore, we hypothesize that for flight cadets, higher perceived social support can reduce the risk of internet addiction. The mechanism may involve two factors: first, by affecting sleep status, and second, by affecting psychological resilience.

Insomnia is one of the direct reflections of stress affecting physical and mental health (16, 17). Flight cadets have strict training schedules, and once under excessive pressure, they may experience insomnia symptoms such as difficulty falling asleep and unstable sleep. Sleep problems have a bidirectional relationship with internet use: excessive internet use disrupts sleep, while poor sleep may prompt individuals to rely excessively on the internet as a compensatory relaxation method (18). Research indicates that internet addicts report significantly higher rates of insomnia and poor sleep quality (19, 20). Among college students, internet addiction is positively correlated with insomnia severity, and both jointly predict negative outcomes such as depression and anxiety (21). Social support may indirectly affect internet addiction by influencing sleep: adequate support helps alleviate anxiety and promote sleep (22, 23), thereby reducing the need to relieve stress through internet use. This shows that insomnia may play a mediating role between social support and maladaptive behaviors—insufficient social support → increased stress and worsened sleep → individuals may resort to excessive internet use to cope, forming a vicious cycle. Therefore, we hypothesize that among flight cadets, insomnia mediates the relationship between perceived social support and internet addiction: cadets with low perceived support are more prone to insomnia, which in turn leads to internet addiction tendencies.

On the other hand, psychological resilience, as an important internal protective personality trait, refers to an individual’s ability to adapt positively and recover quickly when facing stress and adversity (24, 25). Individuals with high psychological resilience typically have better emotional regulation and problem-coping abilities, and are therefore less likely to engage in maladaptive behaviors such as internet addiction. Research has found that psychological resilience is negatively correlated with adolescent internet addiction. For example, Ataman-Bor et al. (2025) showed that adolescents with stronger psychological resilience had significantly lower levels of internet addiction (26). Perceived social support is one of the social environmental factors that enhance psychological resilience: continuous support helps individuals establish a sense of security and self-efficacy, thereby cultivating stronger resilience. Öztürk et al. (2021) pointed out that improving college students’ psychological resilience is an effective way to reduce internet addiction behaviors (27). Similarly, for flight cadets in high-pressure training environments, support from instructors, peers, and family is expected to enhance their psychological resilience, helping them cope with stress in positive ways rather than escaping to the virtual internet for relief. Therefore, we hypothesize that psychological resilience mediates the relationship between perceived social support and internet addiction: higher support levels can promote resilience development, thereby reducing the probability of internet addiction occurrence.

It is worth noting that there is also an association between insomnia and psychological resilience (28, 29). Long-term insomnia not only affects cognition and emotions but also weakens an individual’s psychological resources for coping with adversity, namely reducing psychological resilience. Wang et al. (2020) found that higher resilience scores predicted overall better sleep quality and shorter sleep latency (30). Therefore, within a multiple mediation framework, insomnia and psychological resilience may not be independent of each other but may constitute a chain mediation process: insufficient social support induces insomnia, poor sleep damages psychological resilience, which in turn increases the risk of internet addiction. Previous research in other populations has provided similar evidence. For example, Cui and Chi’s study on adolescents during the pandemic found that perceived social support could reduce internet addiction through the chain path of “improving psychological resilience → reducing PTSD symptoms” (15). Similarly, we have reason to hypothesize that for flight cadets: the chain mediation path of perceived social support → insomnia → psychological resilience → internet addiction is valid.

**Figure 1:**
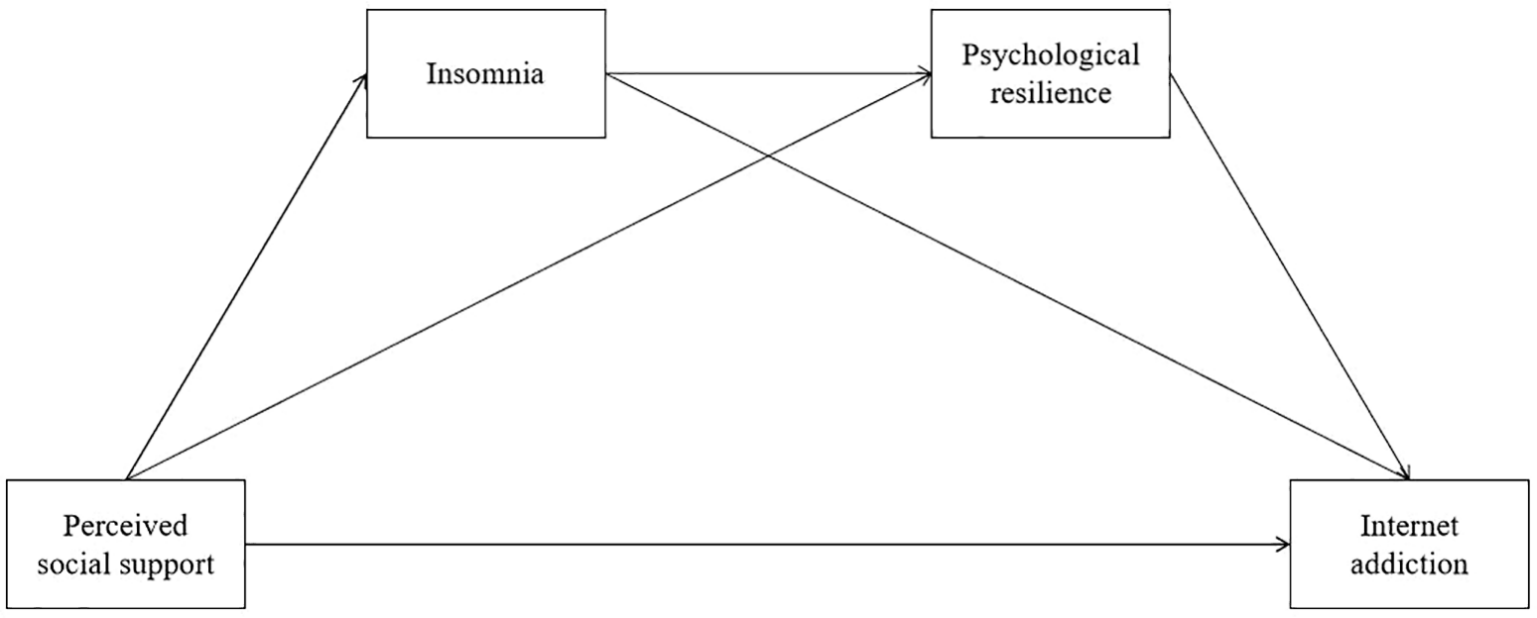
Hypothetical diagram of the chain mediation model.

In summary, this study aims to construct a chain mediation model including insomnia (M_1_) and psychological resilience (M_2_) to explore the mechanism by which perceived social support (X) affects internet addiction (Y) among flight cadets (**Figure 1**). The innovation of this study lies in placing sleep and resilience factors in the same model to examine their sequential mediation effects. This not only helps deepen understanding of the causes of internet addiction among flight cadets but also provides multi-level insights for interventions targeting this group: focusing on both the creation of external support environments and attention to sleep management and resilience cultivation, thereby preventing internet addiction comprehensively. Based on existing literature and theoretical analysis, we propose the hypotheses showed in **Table 1**.

**Table 1:** Summary of study hypotheses.

Hypothesis	Type	Pathway	Expected relationship
H1	Bivariate Correlations	PSS ↔ Insomnia, PSS ↔ Resilience, PSS ↔ IA, Insomnia ↔ IA, Resilience ↔ IA, Insomnia ↔ Resilience	Significant pairwise correlations among all variables
H2	Single Mediation	PSS → Insomnia → IA	Negative indirect effect
H3	Single Mediation	PSS → Resilience → IA	Negative indirect effect
H4	Chain Mediation	PSS → Insomnia → Resilience → IA	Negative indirect effect

PSS, Perceived Social Support; IA, Internet Addiction; → indicates hypothesized directional relationship.

## Materials and methods

### Participants and procedure

Using convenience sampling, 1218 enrolled flight cadets from a civil aviation flight college in China were recruited as participants. All participants in this sample were male, reflecting the predominantly male composition of civil aviation flight cadet programs in China, with ages relatively concentrated in age (17–23 years, M = 18.80 years, SD = 0.79). All participants were informed of the research purpose and provided informed consent. The survey was conducted using anonymous self-report questionnaires. The survey questionnaire was in electronic form, with links distributed through college teaching administrators. Participants voluntarily completed the questionnaire under unified instructions, taking an average of about 15 minutes. After eliminating data with missing key items, a total of 1218 valid samples were obtained. The studies involving humans were approved by Ethics Committee of the Civil Aviation Flight University of China (approval number: 20240014).

### Instruments

A demographic questionnaire was developed by the research team to collect background information from participants. The questionnaire items were designed based on standard demographic assessment practices in Chinese educational research. The variables assessed included: (1) Age and grade level (freshman or sophomore). Participants were limited to freshman and sophomore cadets because these early years represent a critical foundation period before flight training, and junior/senior cadets are dispersed to flight training bases for intensive flight training, making unified survey administration challenging; (2) Family residence (urban or rural), based on the Chinese National Bureau of Statistics’ urban-rural classification system; (3) Only child status (yes/no); (4) Smoking and drinking habits (smoking only, drinking only, both, or neither). These demographic variables were selected based on previous research indicating their potential influence on internet addiction and mental health outcomes among Chinese college students.

The Perceived Social Support Scale (PSS-12) was used to assess flight cadets’ subjectively experienced level of social support (31). The scale contains 12 items across three dimensions of family, friends, and significant others, with each item scored on a 7-point Likert scale (1=“strongly disagree”, 7=“strongly agree”). The sum of all item scores represents the total perceived social support score, ranging from 12-84, with higher scores indicating stronger subjective support. The Cronbach’s α coefficient measured in this study was 0.97, indicating good reliability.

The Athens Insomnia Scale (AIS) was used to measure participants’ insomnia symptom severity over the past month (32). The AIS contains 8 items covering difficulty falling asleep, nighttime awakening, early awakening, total sleep time, overall sleep quality, daytime mood, daytime function, and daytime drowsiness. Each item is scored 0-3 (0=no problem, 3=severe problem), with total scores ranging from 0-24, where higher scores indicate more severe insomnia. In this study, we used the Chinese version of the AIS scale for assessment (e.g., items such as “how long to fall asleep after lights out”, “whether waking up multiple times at night”). In this sample, the internal consistency reliability α coefficient of the AIS scale was 0.82.

The Chinese version of the Connor-Davidson Resilience Scale (CD-RISC) was used to assess flight cadets’ psychological resilience level (33). We used the original scale compiled by Connor and Davidson, containing 25 items covering multiple aspects including coping with change, goal achievement, self-efficacy, sense of humor, and perseverance (example items such as “I can adapt to change”, “I don’t give up easily when facing setbacks”). Each item is scored on a 5-point scale (0=“never”, 4=“almost always”). Higher scores indicate stronger psychological resilience. The α coefficient of this scale in this study was 0.96, showing extremely high internal consistency.

The Chinese Internet Addiction Scale-Revised (CIAS-R) was used for internet addiction measurement (34). CIAS-R was developed by Chen et al., including 5 dimensions: compulsive use, withdrawal symptoms, tolerance, interpersonal and health problems, and time management problems, with 19 items total, each scored on a 4-point scale (1=“completely disagree”, 4=“completely agree”). This study used the total score to measure the degree of internet addiction, with scores ranging from 19-76, where higher scores represent more severe internet addiction tendencies. Chen et al. reported that this scale has good reliability and validity among Chinese college students. The CIAS-R total scale α coefficient in this sample was 0.97, indicating good reliability.

### Statistical analysis

SPSS 24.0 was used for statistical analysis. First, descriptive statistics and Pearson correlation coefficients of the main variables were calculated to preliminarily understand the relationships between variables. Subsequently, Hayes’ PROCESS macro Model 6 was used to test the chain mediation effects of insomnia (M_1_) and psychological resilience (M_2_). In the model, perceived social support served as the independent variable (X), internet addiction as the dependent variable (Y), with insomnia and psychological resilience sequentially entered as mediators. We simultaneously controlled for the effects of background variables such as gender and age. The bias-corrected nonparametric percentile Bootstrap method (5000 repeated samples) was used to estimate the 95% confidence interval (CI) of the mediation effects, with mediation effects considered significant if the CI did not contain 0. All tests used two-tailed testing with a significance level set at 0.05.

## Results

### Descriptive statistics and correlation analysis

**Table 2** summarizes the demographic characteristics of the participants. The 1,218 civil aviation flight cadets had a mean age of 18.80 ± 0.79 years, ranging from 17 to 23 years old. Regarding grade level, 718 participants (58.9%) were freshmen, and 500 (41.1%) were sophomores. In terms of family residence, 1,032 participants (84.7%) were from urban areas, while 186 (15.3%) were from rural areas. Concerning only child status, 661 participants (54.3%) were only children, and 557 (45.7%) had siblings. Regarding smoking or drinking habits, 10 participants (0.8%) reported smoking only, 32 (2.6%) reported drinking only, 33 (2.7%) reported both habits, and 1,143 (93.8%) reported neither smoking nor drinking habits.

**Table 2:** Descriptive statistics of participant characteristics (N = 1218).

Variable	Category	n (%)/M ± SD
Age (years)	–	18.80 ± 0.79
Grade level		
	Freshman	718 (58.9%)
	Sophomore	500 (41.1%)
Family residence		
	Urban	1032 (84.7%)
	Rural	186 (15.3%)
Only child status		
	Yes	661 (54.3%)
	No	557 (45.7%)
Smoking or drinking habits		
	Smoking	10 (0.8%)
	Drinking	32 (2.6%)
	Both	33 (2.7%)
	Neither	1143 (93.8%)

**Table 3** presents the means, standard deviations, and correlation coefficients between variables. Results show that all variables are significantly correlated with each other (p < 0.01). Specifically, perceived social support is significantly negatively correlated with internet addiction (r = -0.34), indicating that the more social support received, the lower the degree of internet addiction. Perceived social support is significantly negatively correlated with insomnia (r = -0.26), suggesting that cadets with high social support have fewer insomnia symptoms. Perceived social support is significantly positively correlated with psychological resilience (r = 0.65), meaning cadets with stronger perceived support have higher psychological resilience. Insomnia is significantly positively correlated with internet addiction (r = 0.30), indicating that cadets with severe insomnia are more likely to develop internet addiction tendencies. Psychological resilience is significantly negatively correlated with internet addiction (r = -0.44), meaning populations with high psychological resilience have lower internet addiction risk. Additionally, insomnia is significantly negatively correlated with psychological resilience (r = -0.28), suggesting that poor sleep may damage an individual’s psychological resilience. These correlation results support our hypothetical premises: social support, sleep, psychological resilience, and internet addiction are closely related, satisfying the conditions for conducting multiple mediation analysis.

**Table 3:** Means, standard deviations, and correlation coefficients of variables (N = 1218).

Variables	M	SD	1	2	3	4
1.Perceived social support	73.67	10.79	–			
2.Insomnia	10.72	2.33	–0.26***	–		
3.Psychological resilience	80.38	15.06	0.65***	–0.28***	–	
4.Internet addiction	28.40	9.49	–0.34***	0.30***	–0.44***	–

***p < 0.001. M, mean; SD, standard deviation.

### Chain mediation effect testing

After controlling for background variables such as age, grade, family residence, only child status, smoking history, and drinking history, we conducted regression analysis on the chain mediation model shown in **Figure 1**, with results listed in **Table 4**. First, the regression coefficient of perceived social support on insomnia is significantly negative (β = -0.25, t = -9.45, p < 0.001), indicating that the higher the level of social support, the lower the degree of insomnia among flight cadets. Second, in the regression predicting psychological resilience, the effect of perceived social support is significantly positive (β = 0.62, t = 27.94, p < 0.001), and the effect of insomnia is significantly negative (β = -0.12, t = -5.23, p < 0.001). This indicates that the more perceived social support, the stronger the cadets’ psychological resilience; if severe insomnia exists, cadets’ psychological resilience will decrease, meaning insomnia has a significant negative prediction on psychological resilience. Finally, in the regression equation predicting internet addiction, the direct effect of perceived social support is negative and reaches a significant level (β = -0.07, t = -1.98, p < 0.05), indicating that social support still has a direct inhibitory effect on internet addiction after controlling for mediating variables; the effect of insomnia on internet addiction is positive and significant (β = 0.18, t = 6.76, p < 0.001), and the effect of psychological resilience is negative and significant (β = -0.34, t = -10.12, p < 0.001). This indicates that when considering social support and psychological resilience simultaneously, higher insomnia severity significantly increases internet addiction risk, while higher psychological resilience significantly reduces internet addiction tendencies. The above regression results indicate that the effect of perceived social support on internet addiction has both direct paths and is indirectly realized through insomnia and psychological resilience.

**Table 4:** Regression analysis among variables in the chain mediation model.

Outcome variable	Predictor variable	*R*	*R* ^2^	*F*	*β*	*t*
Insomnia	Perceived social support	0.31	0.10	21.76^**^^*^	-0.25	-9.45^**^^*^
Psychological resilience	Perceived social support	0.67	0.45	139.37^**^^*^	0.62	27.94^**^^*^
	Insomnia				-0.12	-5.23^**^^*^
Internet addiction	Perceived social support	0.49	0.24	47.30^**^^*^	-0.07	-1.98^*^
	Insomnia				0.18	6.76^**^^*^
	Psychological resilience				-0.34	-10.12^**^^*^

*p<0.05, ***p < 0.001. The demographic variables are controlled as covariates.

**Figure 2:**
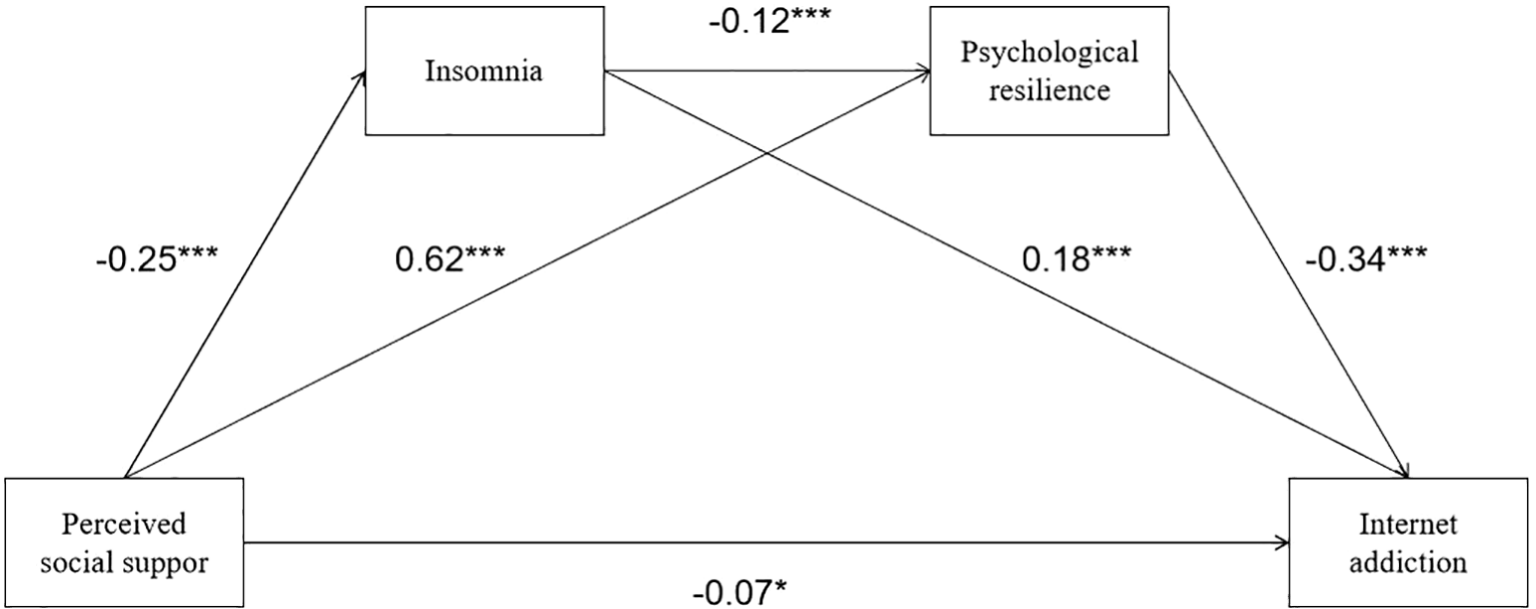
Path coefficient diagram of the chained mediation model. *p<0.05, ***p<0.001.

To further test the significance of mediation effects, we used the nonparametric Bootstrap method to calculate the indirect effect values and confidence intervals of each path, with results shown in **Table 5**. When the 95% confidence interval does not contain 0, it indicates that the mediation effect is significant. **Table 5** shows: (1) The effect of perceived social support indirectly affecting internet addiction through the single mediating variable “insomnia” is -0.05, 95% CI is [-0.07, -0.03], not containing 0, indicating that this indirect effect is significant. In other words, perceived social support reduces insomnia, and insomnia severity positively predicts internet addiction risk, thereby forming a significant indirect path. (2) The indirect effect of perceived social support through the single mediation of “psychological resilience” is -0.21, 95% CI is [-0.26, -0.17], also significant. This is the path with the largest effect size among all mediation paths, demonstrating the prominent position of psychological resilience in the protective role of social support. (3) The indirect effect of perceived social support through the “insomnia→psychological resilience” chain mediation is -0.01, 95% CI is [-0.02, -0.01], with a small effect size but the confidence interval does not contain 0, reaching a significant level. This means there exists such a sequential mechanism: insufficient social support triggers sleep problems, which then weakens psychological resilience, ultimately increasing the risk of internet addiction. (4) The total effect of perceived social support on internet addiction is -0.34 (95% CI = [-0.39, -0.28]), and the direct effect is -0.07 (95% CI = [-0.13, -0.01]). It can be seen that after including mediators, the significance of the direct effect of social support is weakened, but there is still a portion of direct effect not explained by mediating variables (**Figure 2**).

**Table 5:** Analysis of the mediating effect of insomnia and psychological resilience.

Pathway	Effect size	Standard error	Boot CI LL	Boot CI UL	Relative mediation effect %
Direct effect	-0.07	0.03	-0.13	-0.01	20.59%
Indirect effect 1	-0.05	0.01	-0.07	-0.03	14.71%
Indirect effect 2	-0.21	0.02	-0.26	-0.17	61.76%
Indirect effect 3	-0.01	0.01	-0.02	-0.01	2.94%
Total mediation effect	-0.34	0.03	-0.39	-0.28	100%

Indirect effect 1: Perceived social support → Insomnia → Internet addiction. Indirect effect 2: Perceived social support → Psychological resilience → Internet addiction. Indirect effect 3: Perceived social support → Insomnia → Psychological resilience → Internet addiction. The demographic variables are controlled as covariates.

## Discussion

This study examined how perceived social support affects internet addiction tendencies among civil aviation flight cadets through two psychological processes of insomnia and psychological resilience, and the results supported our four hypotheses. The main findings include: ① The higher the perceived social support of flight cadets, the lower their level of internet addiction;② Perceived social support can indirectly reduce internet addiction both by reducing insomnia severity and by improving psychological resilience; ③ More importantly, perceived social support can also exert its effect through the chain mediation path of “reducing insomnia→improving psychological resilience”. In brief, when flight cadets perceive adequate social support, they are less likely to experience insomnia and have stronger psychological resilience, thus being less prone to internet addiction; conversely, lack of social support may trigger sleep problems and weaken psychological resilience, thereby increasing the risk of internet addiction.

First, this study verified the direct protective effect of perceived social support on internet addiction. This result is consistent with previous research on college students and adolescents (35). Social support, as emotional and resource support, can help flight cadets alleviate training pressure and loneliness (36). When cadets feel understanding and help from instructors, classmates, and family, they gain a stronger sense of security and belonging, thus having less need to seek comfort or escape reality through internet addiction. This finding also confirms the view of the protective-risk model: social support as an external protective factor can directly combat the adverse outcome of internet addiction, reducing its probability of occurrence overall (10).

Second, the mediating role of insomnia between social support and internet addiction was supported. Previous research has demonstrated that social support can mediate the relationship between internet addiction and sleep quality (23), and meta-analytic evidence shows moderate pooled associations between sleep quality, insomnia and stress in undergraduate students (37). The indirect effect of social support on internet addiction through insomnia in our study demonstrates a specific pathway consistent with these findings: when flight cadets lack emotional support and experience increased stress, they are more likely to develop sleep difficulties, which in turn increases their vulnerability to internet addiction. Research during COVID-19 found that individuals with high social support had a 52% lower risk of poor sleep quality compared to those with low perceived social support (38). This mechanism is particularly relevant for flight cadets who face strict training schedules and high-pressure environments. Our findings extend this evidence to the specific context of aviation training, where irregular schedules may exacerbate sleep-related vulnerabilities. Among college students, insomnia has been shown to partially mediate associations between psychosocial factors (such as interpersonal stress) and mental health outcomes (39), suggesting that sleep disturbances may serve as a mechanism through which social and environmental stressors influence behavioral outcomes. The pathway from social support to internet addiction through insomnia aligns with the stress-vulnerability model, which posits that protective factors can buffer against stress-related outcomes. Longitudinal research has demonstrated stress-buffering effects of social support when measured prospectively over time, providing theoretical support for the protective role of social support observed in our mediation model (40).

Third, the mediating role of psychological resilience is even more prominent. Our data show that a considerable portion of the effect of social support on internet addiction is exerted through psychological resilience. This means that cadets with strong social support often also have stronger psychological resilience, and high psychological resilience makes them more resilient when facing pressure, less likely to cope through maladaptive means (such as internet addiction) (41). Psychological resilience allows individuals to “bounce back” in adversity, while internet addiction can be viewed as a manifestation of failing to properly cope with stress and emotional dysregulation. Previous research has found similar patterns in other high-pressure populations: for example, social support is positively correlated with psychological resilience among military personnel (42). For flight cadets, continuous training pressure and strict requirements may cause some to feel frustrated or burned out, but if they possess stronger psychological resilience, they are more likely to adopt positive coping strategies (such as seeking help, physical exercise) rather than escaping to the internet. Social support precisely cultivates and enhances this resilience: it provides emotional support and problem-solving resources, making cadets believe they are not fighting alone, thus having more confidence and ability to overcome challenges. For example, when flight training encounters bottlenecks, encouragement and guidance from instructors and mutual assistance from classmates can improve cadets’ frustration tolerance, allowing them to pull themselves together and continue working hard, rather than becoming addicted to the internet to escape reality due to frustration. This shows that improving psychological resilience is an important mediating link in the effect of social support.

Finally, the existence of chain mediation effects reveals the sequentiality and association between insomnia and psychological resilience in the process. Although viewed separately, insomnia and resilience each play mediating roles, they are not isolated. Our data indicate that low social support triggers a “domino effect” of problems: first being unable to sleep well (insomnia), then due to long-term fatigue and sleep deprivation, psychological toughness is also weakened, ultimately falling into the danger of internet addiction (27). This can be explained by the positive correlation between sleep quality and psychological resilience (43, 44). Good sleep quality enhances the brain’s emotional regulation function, making individuals less likely to experience stress and more likely to maintain optimism and perseverance. Therefore, when lacking social support, flight cadets on one hand experience subjective pressure and on the other hand lack external channels for relief, which often manifests as sleepless nights; and persistent poor sleep depletes psychological resources, making them more vulnerable in the face of setbacks. Taken together, this chain process exacerbates dependence on the internet. Although the chain effect size is relatively small, possibly indicating that for most people, the effect of social support on psychological resilience does not necessarily need to be realized through sleep, and the two mediation paths are more parallel. However, the significance of the chain path reminds us to pay attention to the significance of sleep-resilience interaction in interventions: simply improving social support or cultivating resilience may not be enough; if cadets are always in a state of sleep deprivation, improvement in resilience may be difficult to fully exert its effect. Therefore, in practice, a two-pronged approach should be taken—both helping cadets improve sleep (such as teaching sleep hygiene knowledge, adjusting training schedules) and cultivating psychological resilience (such as stress management training, frustration education), with the two complementing each other to more effectively prevent maladaptive coping behaviors such as internet addiction.

This study integrates stress-related physiological factors (sleep) and psychological factors (resilience) into the examination of internet addiction formation mechanisms, enriching the theoretical perspective of internet addiction research. First, the results support the applicability of the stress-vulnerability model and protective-risk model to the issue of internet addiction among flight cadets: social support and psychological resilience play protective roles, while insomnia can be viewed as a vulnerable link in stress’s impact on the body. Second, our research highlights the value of multiple mediation chains in explaining complex behavioral problems. Compared to single mediation models, chain mediation models reveal more refined sequences and mechanisms of action between variables. Additionally, this study tested addiction mechanisms slightly different from ordinary students in a high-pressure training group, providing new evidence for the theoretical universality of internet addiction. Our findings indicate that in disciplined and tightly scheduled flight cadets, sleep as a physiological-psychological crossover factor cannot be ignored; it and psychological resilience together link the protective effects of social support, providing ideas for future integration of physical and mental health factors.

The results of this study have important practical implications specifically for civil aviation flight training contexts. First, aviation colleges should establish multi-level support systems tailored to the quasi-military training environment: structured peer mentoring programs that balance the inherent competition in flight training with collaborative learning, instructor training to recognize psychological distress beyond technical evaluation, and family liaison programs that maintain connections despite geographic separation and strict schedules. Second, sleep management is critical given both flight safety implications and insomnia’s mediating role: colleges should optimize training schedules (rotating early flight assignments, implementing mandatory rest periods between intensive phases), integrate sleep hygiene education as an essential professional competency for future irregular schedules, and enforce dormitory policies that limit late-night internet access while improving sleep environments. Third, resilience training must address aviation-specific stressors through simulation-based stress inoculation training, failure reframing workshops where senior pilots share their training setbacks, and mindfulness techniques applicable during pre-flight stress. Finally, regular screening using validated instruments (such as CIAS-R) should identify at-risk cadets for targeted multi-component interventions addressing the specific pathways identified: connecting isolated cadets with peer support, providing cognitive-behavioral therapy for insomnia (CBT-I), implementing structured internet use management alongside diverse recreational alternatives, and cultivating a safety-focused wellness culture.

This study also has some limitations. First, the study used a cross-sectional design, thus unable to strictly infer causal relationships; future studies could consider longitudinal tracking or experimental intervention methods to more powerfully verify causal chains. Second, the sample consisted entirely of male cadets from a single flight college, which limits the generalizability of findings to female populations and other institutional contexts. Gender differences in internet addiction patterns, social support utilization, and psychological resilience suggest that the identified mediation pathways may operate differently across genders and warrant further investigation in gender-diverse samples. Additionally, this study only examined two mediating variables of insomnia and psychological resilience, while mechanisms affecting internet addiction in reality may be more complex. Future research could construct more complex models including emotional symptoms, or introduce moderating variables (such as gender, personality traits) to examine differences in this model across different groups.

## Conclusion

In summary, this study reveals the mechanism by which perceived social support reduces internet addiction among civil aviation flight cadets by decreasing insomnia and improving psychological resilience. Cadets with high social support have significantly lower internet addiction tendencies, and this protective effect is partly due to their better sleep and stronger psychological resilience; low social support may lead to poor sleep and insufficient resilience, which together trigger higher internet addiction risk. This finding expands our understanding of the causes of internet addiction in high-pressure pre-professional groups and also emphasizes the importance of intervention from multiple angles of social support-sleep-resilience. For colleges cultivating flight talents, efforts should be made to provide cadets with a good support environment, pay attention to sleep health and psychological quality improvement, thereby preventing and reducing the occurrence of maladaptive behaviors such as internet addiction.

## Data Availability

The raw data supporting the conclusions of this article will be made available by the authors, without undue reservation.
